# Misdiagnosis of a Massive and Advanced Seminoma as an Inguinal Hernia: A Case Report

**DOI:** 10.7759/cureus.31001

**Published:** 2022-11-02

**Authors:** Sarya Swed, Abdulqadir J Nashwan, Mohammad Y Naal, Weaam Ezzdean, Amine Rakab

**Affiliations:** 1 Medicine, Aleppo University, Aleppo, SYR; 2 Nursing Department, Hamad Medical Corporation, Doha, QAT; 3 Urology Department, Aleppo University Hospital, Aleppo, SYR; 4 Urology Department, Ibn Al-Nafees Hospital, Damascus, Syria, Damascus, SYR; 5 General Internal Medicine, Weill Cornell Medicine-Qatar, Education City, QAT

**Keywords:** misdiagnosing, urologic oncology, inguinal hernia, unusual presentation, seminoma

## Abstract

Misdiagnosing a seminoma as an inguinal hernia is an uncommon medical concern. A 22-year-old male presented with an enlarged, painless lump in the right scrotum above the right testis. He had previously been given a diagnosis of inguinal hernia by a general practitioner in a rural area two years ago. Pelvic trauma had never occurred before. Scrotal ultrasonography revealed that his right testis was hypoechoic, asymmetrical, and solidly enlarged. A radical orchiectomy was done to remove the tumor. A histological examination of the mass confirmed the diagnosis of classical seminoma. Six months later, the patient had no complications and was in good condition. General practitioners must be more mindful of this tumor, particularly its presentation, since seminoma tumors might be misdiagnosed as an inguinal hernia. This is necessary to preserve the reproductive organs and achieve good outcomes.

## Introduction

Seminomas are malignant germ cell tumors that typically affect the testis, although they may also occur less often in the mediastinum, retroperitoneum, or other extra-gonadal locations. The condition is very uncommon, with an incidence rate of just eight to 10 cases per 100,000 males per year in the nations of northern Europe. The incidence of this neoplasm is highest in young men between the ages of 20 and 45, in contrast to the majority of other cancers [1]. Most testicular cancer cases present with a painless, unilateral testicular scrotal lump, and are usually identified incidentally during the clinical examination or may be discovered post scrotal trauma. In addition, the most common risk factors for testicular cancer are germ cell neoplasia in situ, hypogonadism, undecadent testis, family history of prostate cancer, testicular atrophy, and testicular calcifications. However, these hernias may also delay detection in rare instances when testicular cancer is mistakenly diagnosed as an inguinal hernia [2]. One-quarter of patients with testicular cancer get an incorrect diagnosis, and the typical length of time that occurs between the initial signs and symptoms of the disease and the beginning of decisive therapy is three to six months [2]. The treatment options for seminoma depend mostly on the type and stage. They may include careful surveillance, surgical removal of the testis, radiation therapy, radical inguinal orchiectomy, chemotherapy, and retroperitoneal lymph node dissection. This is an uncommon case of a seminoma misdiagnosed as a right inguinal hernia by a general practitioner in a remote rural area where the left testicle was normal. The tumor's late staging resulted in losing the right testicle.

## Case presentation

**Figure 1:**
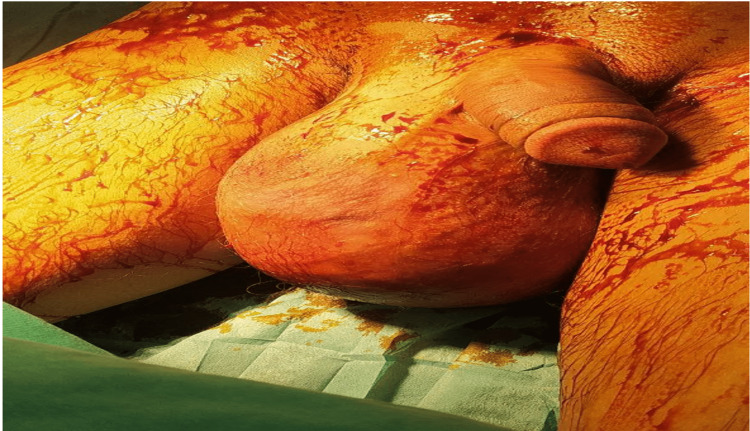
Enlargement of the right scrotum

A 22-year-old man presented to the urology clinic department with increased size, painless palpable, and a massively large mass in the right scrotum superior to the right testis. It was previously diagnosed as an inguinal hernia for two years by a general practitioner in a rural area. He was a student and a non-smoker; his medical history revealed no significant points except for this mass, which was misdiagnosed as an inguinal hernia two years ago, with no history of pelvic trauma. We detected a massive painless mass impermeable to light occupying the scrotum's right side with a heaviness sensation over the affected area but without tenderness. There was no palpable inguinal lymph node or a change in the scrotum color (Figure 1).

**Figure 2:**
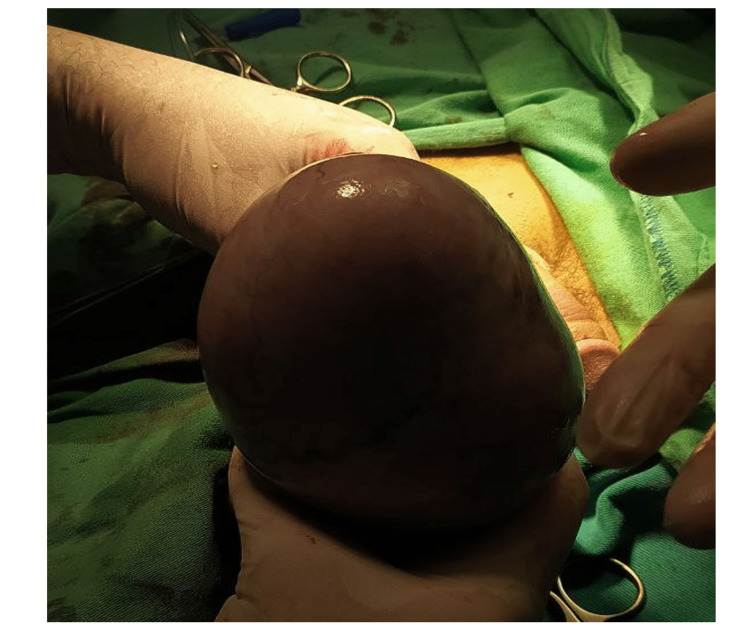
The removed mass showing the massive size

Examination of other systems did not reveal abnormalities. The results of laboratory blood tests, including those for cell blood counts, kidney, and liver function, were all within normal ranges. A scrotal ultrasound revealed a hypoechoic asymmetrical solid enlargement measuring nearly 12 cm of the right testis and a normal-shaped left testis. Laboratory test results showed serum alpha-fetoprotein levels of 0.5 IU/ml (normal range: 0-40 IU/ml), the beta subunit of human chorionic gonadotropin of 3.9 IU/ml (normal range: less than 5 mIU/mL), and lactate dehydrogenase of 1665 U/l. A pelvic multi-slice CT scan did not reveal retroperitoneal metastases or enlarged lymph nodes, and the testicles and scrotum were enlarged, but there were no signs of inflammation. The tumor, node, and metastasis (TNM) classification of this tumor was T3N0M0. In light of the previous data, the primary diagnosis was of a testicular tumor. Radical orchiectomy to remove the mass was the treatment of choice. In this case, a right inguinal approach was utilized to perform a radical orchiectomy of the right testis, in which a large mass of 13-14 cm was removed successfully (Figure 2).

**Figure 3:**
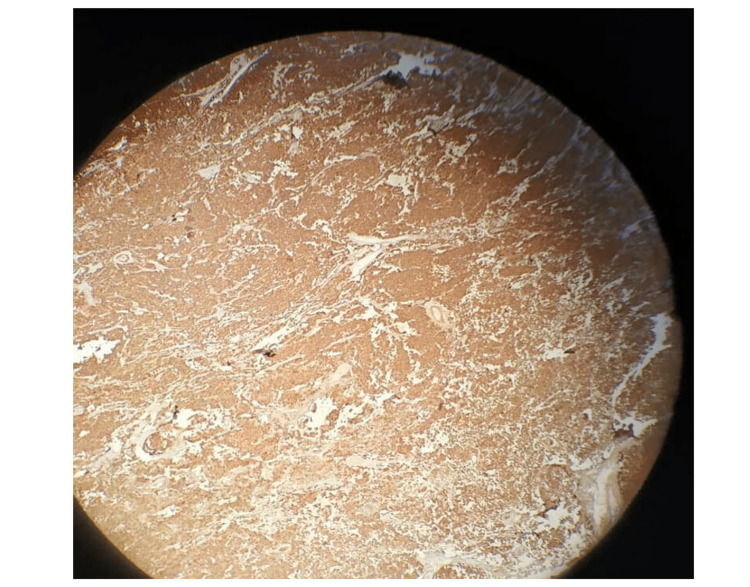
PLAP immunohistochemical stain showing strong membranous positivity in seminoma cells PLAP: placental alkaline phosphatase

The only obstacle faced during the surgery was the massive tumor size, which was appropriately managed. No complications or significant bleeding occurred during the surgery. Paracetamol, ceftriaxone, and sulbactam were administered intravenously after the operation. A right radical orchiectomy with the measurement of 14 x 13 x 8 cm revealed an attachment to the spermatic cord measuring 6 cm; the cut surface showed a substantial white tumor mass filling the full testis that measured 13 cm. However, the microscopic picture revealed nodules of typical rounded cells with hyperchromatic, large nuclei, prominent nucleoli, and clear cytoplasm, surrounded by the trabeculae infiltrated by lymphocytes forming a sheet-like pattern or confluent multinodular pattern. Intense immune reactivity with placental alkaline phosphatase (PLAP) was positive (Figure 3), confirming the diagnosis of classical seminoma. The patient was discharged after 24 hours without any complications. The oncologist suggested that radiation and chemotherapy after surgery are not recommended. The postoperative follow-up approach should be made instead, which included hematological and standard laboratory tests, tumor markers, a multi-slice CT scan, and bone scintigraphy every six months for one year after surgery. These diagnostic tests were repeated after 12 months and found within normal limits without evidence of retroperitoneal or bone metastases.

## Discussion

We present a rare case of a patient with seminoma who presented with an inguinal hernia, whose diagnosis was made inadequately by a general practitioner from a remote region. The differential diagnosis of testicular malignancies comprises several conditions, such as epididymitis, hematocele, hydrocele, spermatocele, granulomatous orchitis, varicocele, and inguinal hernia. In a few cases, the illness may be misdiagnosed as a less severe condition, treatment may be delayed, and clinical examinations performed by untrained general practitioners who lack the resources to make the right diagnosis may worsen the outcome. It is also considered a major medical concern during clinical treatment that has caused the loss of the right testicle in this patient. In this case, we discussed a few other cases reported in the literature associated with uncommon presentations of testicular cancers, such as

Jo et al. [3] reported an unusual case of huge spermatic cord lipoma as an inguinal hernia. The patient, a 33-year-old man, had undergone laparoscopic right herniorrhaphy and came with right scrotal enlargement with no postoperative clinical benefit. Physical examination revealed a non-tender lump in the right scrotum, which was the size of an adult fist. A club-shaped increased fatty component of the spermatic cord was seen on abdominopelvic CT in the right inguinal canal and right scrotal sac. The surgical results via the inguinal incision revealed no herniation or attachment to the peritoneum. It was necessary to remove the right vas deferens since it was impossible to identify them. Spermatic cord lipoma was detected through histopathology. Ben Hadj Alouane et al. [4] presented an unusual case of a 57-year-old man who had been experiencing discrete left flank pain for two months. A large pelvic mass was discovered by a CT scan, coupled with a second retroperitoneal mass that was the cause of left hydronephrosis. The pelvic mass was surgically removed, and histology confirmed the diagnosis of seminoma.

Furthermore, Kazmi et al. [5] described a 54-year-old man with increasing frequency of urination, nocturia, hesitation in urinating, urgency of urination, and a weak stream. After receiving a preliminary diagnosis of benign prostatic blockage, the patient was scheduled for trans-urethral prostate removal (TURP). Intraoperative examinations revealed a benign-appearing prostate. The postoperative course continued without incident, and the patient was subsequently released. Histopathology provided the evidence necessary to conclude the prostate's primary seminoma diagnosis.

The lessons learned from this unfortunate case may include that seminoma should be diagnosed early and adequately with a multi-step procedure that includes a medical history, examination findings, testicular ultrasonography, and medical tests for diagnostic markers. Moreover, we need to improve the medical situation in Syria's rural regions by bringing professionals to train the local general practitioners in medicine and giving them the necessary support and sufficient medical equipment for accurate diagnosis and treatment.

## Conclusions

General practitioners must pay more attention by conducting an adequate clinical examination if they find an inguinal hernia, especially in large sizes. They must also investigate the presence of asymptomatic pelvic cancers such as seminoma to avoid advanced prognosis of the tumors and serious complications (mainly loss of one of the important organs) due to medical misdiagnosis or lack of follow-up. This is especially important when the patients are from remote area in a war zone.
